# Tumor-Associated Macrophages Regulate PD-1/PD-L1 Immunosuppression

**DOI:** 10.3389/fimmu.2022.874589

**Published:** 2022-05-03

**Authors:** Yunzhou Pu, Qing Ji

**Affiliations:** Department of Medical Oncology and Cancer Institute, Shuguang Hospital, Shanghai University of Traditional Chinese Medicine, Shanghai, China

**Keywords:** immune checkpoint inhibitor (ICI), PD-1/PD-L1 axis, immunosuppression, tumor-associated macrophages (TAMs), immune microenvironment

## Abstract

Anti-programmed cell death 1 (PD-1) or anti-PD-ligand (L) 1 drugs, as classic immune checkpoint inhibitors, are considered promising treatment strategies for tumors. In clinical practice, some cancer patients experience drug resistance and disease progression in the process of anti-PD-1/PD-L1 immunotherapy. Tumor-associated macrophages (TAMs) play key roles in regulating PD-1/PD-L1 immunosuppression by inhibiting the recruitment and function of T cells through cytokines, superficial immune checkpoint ligands, and exosomes. There are several therapies available to recover the anticancer efficacy of PD-1/PD-L1 inhibitors by targeting TAMs, including the inhibition of TAM differentiation and re-education of TAM activation. In this review, we will summarize the roles and mechanisms of TAMs in PD-1/PD-L1 blocker resistance. Furthermore, we will discuss the therapies that were designed to deplete TAMs, re-educate TAMs, and intervene with chemokines secreted by TAMs and exosomes from M1 macrophages, providing more potential options to improve the efficacy of PD-1/PD-L1 inhibitors.

## Introduction

Cancer is a worldwide health problem, with an increasing number of confirmed cases and a high mortality (1). Immune escape is one of the most important characteristics of cancers. Tumors reduce immunogenicity as they divide and proliferate, leading to immune escape. Immune checkpoint inhibitors (ICIs) are a new method for tumor immune escape that yields survival benefits for tumor patients. The first ICI that was developed targeted the protein cytotoxic T-lymphocyte antigen 4 (CTLA-4)-ipilimumab (2), which increased survival by 3.7 months in patients with advanced melanoma and boosted the field of cancer treatment. ICIs bind to CTLA-4 or PD-1 and its ligand PD-L1, the key targets related to T-cell activation and exhaustion, and then eliminate immune suppression by tumors.

In the tumor microenvironment (TME), PD-L1 is expressed on the surface of tumors and binds to PD-1 on T cells to resist the killing effect of T cells, ultimately causing tumor immune escape. The application of anti-PD-1/PD-L1 monoclonal antibodies (mAbs) to block the PD-1/PD-L1 signaling pathway has shown excellent antitumor efficacy in a variety of solid tumors (3). However, clinical studies have demonstrated that some patients do not respond to the therapy, and some patients even exhibit tumor recurrence after a period of remission (4). Drug resistance is a crucial factor that determines the efficiency of anti-PD-1/PD-L1 ICIs. Therefore, a deeper understanding of the regulation of the PD-1/PD-L1 axis is essential for the improvement of antitumor immunotherapy.

The mechanisms of resistance to PD-1/PD-L1 blockade mainly include dysfunction or activation disorder of T cells, depletion or reduced infiltration of T cells, and changes in PD-L1 expression (5). The infiltration of T cells in the TME is the precondition of antitumor immunity, while the infiltration of immunosuppressive cells is the premise of tumor immune escape. Tumor-associated macrophages (TAMs) are immunosuppressive cells that induce drug resistance to PD-1/PD-L1 therapy. As one of the most abundant cell types in solid tumors, TAMs contribute to T-cell dysfunction and exhaustion through the secretion of cytokines and metabolic products (6–8) and increase PD-L1 expression in tumor cells and other immunosuppressive cells (9–11). In diagnosed cancers, high macrophage infiltration is often closely related to the occurrence of drug resistance to PD-1/PD-L1 immune suppressants (12–14). Therefore, TAMs have been suggested as important targets to reverse the resistance to anti-PD-1/PD-L1 therapy. In this review, we highlight the recent findings of the suppressive effects of TAMs on PD-1/PD-L1 checkpoint inhibitors. To facilitate precision medicine and expand the target population, we further discuss combination therapies that may improve the efficacy of ICIs targeting PD-1/PD-L1.

## TAMs Modulate the Expression and Functions of PD-1/PD-L1

**Figure 1:**
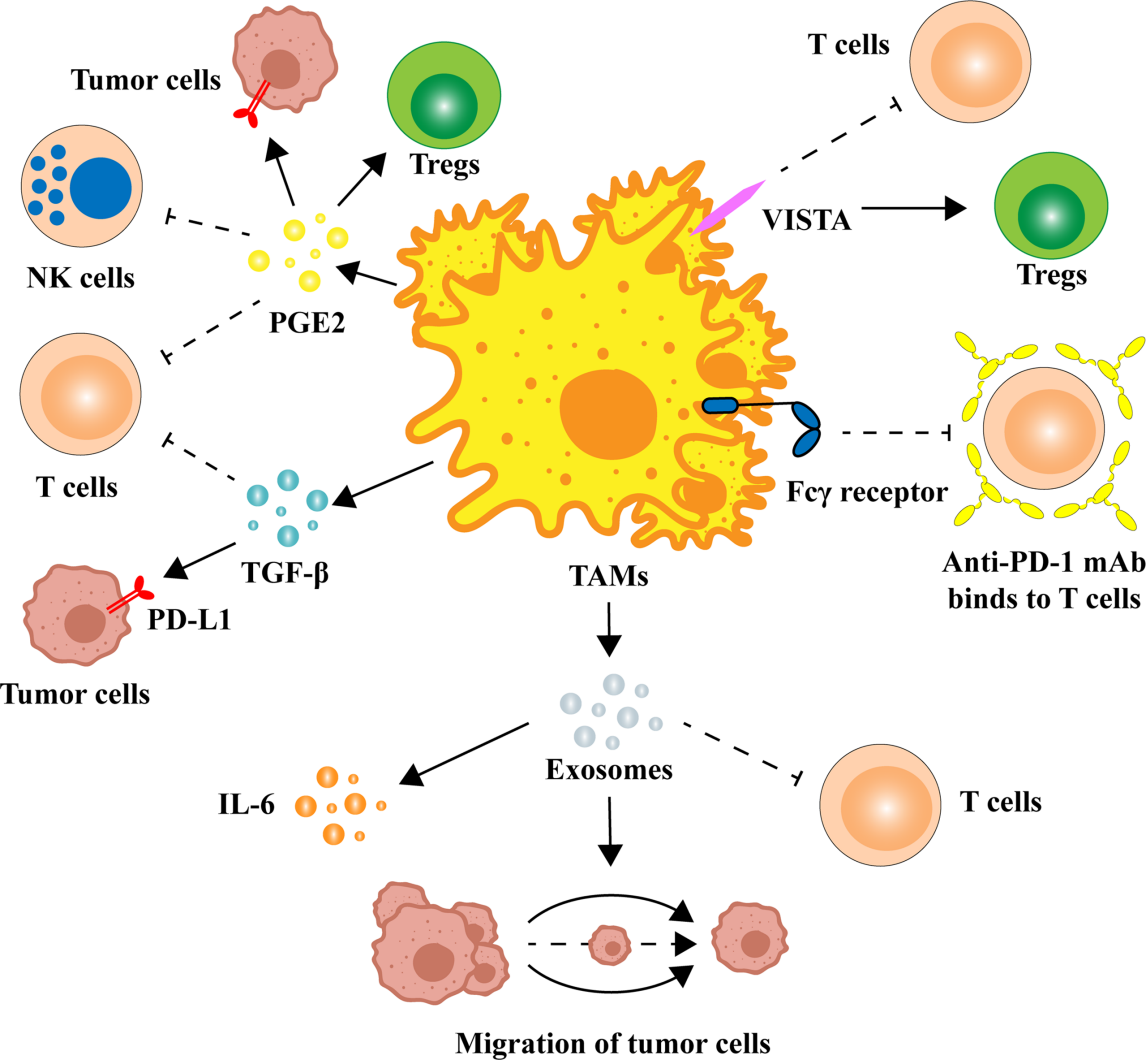
Multiple ways in which TAMs regulate the expression and function of PD-1/PD-L1. TAMs can release a variety of cytokines to alter the TME, such as TGF-β and PGE2. There are also homologous immune checkpoint ligands on the surface of TAMs that can block anti-PD-1/PD-L1 immune efficacy. M2 TAM-derived exosomes are also potentially associated with PD-1/PD-L1 inhibitors. IL, interleukin; mAb, monoclonal antibody; NK cell, natural killer cell; PD-1, programmed cell death 1; PD-L1, programmed cell death ligand-1; PGE2, prostaglandin E2; TAM, tumor-associated macrophage; TGF-β, transforming growth factor-β; Treg, regulatory T cell; VISTA, V-domain Ig-containing suppressor of T-cell activation.

Macrophages have powerful functions in identifying, phagocytosing, and removing bacteria and foreign bodies in the immune system. In the process of tumorigenesis, macrophages evolve, resulting in the properties of TAMs that promote tumor growth (15). Mounting evidence suggests that secretions or exosomes from tumor cells shift the transcriptional program of TAMs from the M1-like phenotype to the M2-like phenotype (16–20). In a variety of cancers, the infiltration of M2 TAMs is significantly related to poor prognosis, tumor progression, and other adverse clinical outcomes (14, 21–24). Moreover, in the process of anti-PD-1/PD-L1 immunotherapy, M2 TAMs can also suppress immunotherapy efficacy by inhibiting T-cell activity and enhancing the expression of PD-L1 in the TME. Specifically, M2 TAMs inhibit the function of PD-1/PD-L1 blockers by secreting anti-inflammatory cytokines and exosomes, increasing superficial immune checkpoint ligands (**Figure 1**).

### Cytokines Secreted by TAMs

TAMs are capable of secreting several cytokines, which mediate tumor-promoting activity and immunosuppression in the TME. Among them, transforming growth factor-β (TGF-β) and prostaglandin E2 (PGE2) are noteworthy due to their strong immunosuppressive effect and high correlation with TAM (25–29).

TGF-β has been shown to affect anti-PD-1/PD-L1 immunotherapy by inhibiting the activation of T-cells and the expression of PD-L1 (30). TGF-β derived from TAMs inhibits T-cell activity by decreasing the expression levels of IFN-γ and Granzyme B (indicating cytotoxic activity) in T cells through phosphorylation of the Smad2/3 protein and the inhibition of mitochondrial respiration (31, 32). The expression of TGF-β was associated with the infiltration of T cells. Tumors with higher expression of TGF-β presented lower infiltration of CD8^+^ T cells. TGF-β can also modulate the function of PD-1/PD-L1 by regulating PD-L1 expression. In solid tumors such as breast cancer, TGF-β can induce the upregulation of PD-L1 in tumor cells and tumor-associated angiogenesis, which may be associated with the accumulation of succinate in tumor cells (11). Increased TGF-β levels in the TME not only promoted T-cell exclusion and accumulation of regulatory T cells (Tregs) (33) but also blocked the acquisition of the Th1 effector phenotype (34). In addition, since TGF-β in the TME originates from a variety of cells (35), immunosuppression induced by crosstalk between these cells should also be noted.

PGE2 can inhibit T-cell activation and function by increasing the expression of PD-L1 (36–38). As a downstream of cyclooxygenase 2 (COX-2), the level of PGE2 in the TME is regulated by the expression of COX-2 and microsomal PGE2 synthase 1 (mPGES1) (39). In bladder cancer, TAMs can increase the expression of PD-L1 in tumor-infiltrating myeloid cells through the COX-2/mPGES1/PGE2 pathway, which leads to the exclusion of CD8^+^ T cells (7). Similarly, PGE2 upregulates PD-L1 expression in ovarian cancer cells by activating the PI3K-AKT-mTOR pathway (40). Moreover, PGE2 can induce the expression of Forkhead Box P3 (Foxp3) to stimulate the differentiation of immunosuppressive Tregs from naïve T cells (41). Of particular note is that therapies targeting PGE2 with NSAIDs or COX-2 inhibitors fail in clinical trials due to global prostaglandin inhibition, which in turn could cause serious side effects (42). Therefore, precision therapy targeting macrophages should be proposed, which may be the next step in reversing drug resistance to PD-1/PD-L1 therapy.

### Ligands Expressed by TAMs

In addition to the expression of PD-L1 on the surface of TAMs, there are also homologous immune checkpoint ligands that can block anti-PD-1/PD-L1 immune efficacy. V-domain Ig-containing suppressor of T-cell activation (VISTA), an immune checkpoint ligand expressed by TAMs (43–46), is an immunosuppressive molecule that reduces T-cell proliferation and cytokine production while sustaining Treg function (46). The expression of VISTA is not only positively correlated with the expression of PD-L1 on the surface of tumor cells but also correlated with the patient’s poor prognosis, pathological grade, and lymph node status (47, 48). In fact, a recent study demonstrated a strong correlation between VISTA expression and tumor infiltration by myeloid cells and PD-1^+^ inflammatory cells (49). Targeting VISTA antibodies can regulate innate immunity and adaptive immunity by promoting T-cell infiltration, thereby slowing tumor growth in mouse cancer models (50). However, VISTA is also highly expressed in hematopoietic and microglial cells (51, 52), and more studies on the systemic responses to VISTA-targeted therapies are needed. We look forward to its future application in combination with anti-PD-1/PD-L1 mAbs.

Moreover, TAMs can prevent the interaction of anti-immune checkpoint mAbs with the targets through the Fcγ receptor present on cell surfaces (53). Indeed, studies have demonstrated that after administration, anti-PD-1 mAb binds to tumor-infiltrating T cells at an early stage but is subsequently captured by TAMs due to the presence of Fcγ receptors, which ultimately leads to drug failure (53, 54). More importantly, activation of Fcγ receptors by an anti-PD-1 mAb results in depletion of activated CD8^+^ T cells *in vitro* and *in vivo*, reducing the therapeutic effect (55). Therefore, the design of FC-null anti-PD-1 mAbs (55) or specific competitive inhibitors is one of the future strategies necessary to block Fcγ receptor-mediated resistance and increase T-cell infiltration.

### Exosomes Derived From M2 TAMs

Exosomes in the TME have been reported as a medium of communication between cells for the occurrence and invasion of tumors (56–58). Exosomes are able to promote the migration of cancer cells through the PI3K-AKT signaling pathway activated by apolipoprotein E (59). The effect of M2 TAM-derived exosomes on drug resistance has also been reported (60), and it has been verified that microRNAs in exosomes are key regulators of resistance to gemcitabine (60). Analogously, miRNAs of M2 TAM-derived exosomes have also been implicated in the regulation of anti-PD-1/PD-L1 immunotherapy. MicroRNA-21 (MiR-21) expression is relatively high in glioma and associated with low infiltration of CD8^+^ T cells. Inhibiting miR-21 in exosomes not only improves the proliferation and cytotoxic activity of CD8^+^ T cells but also reduces the level of TGF-β1, which prevents immune escape of glioma cells (61). In addition, another study showed that the combination of miR-21 deletion and anti-PD-1 treatment demonstrates better antitumor activity than either drug alone (62). Moreover, *in vivo*, miR-155-5p in exosomes secreted by M2 TAMs can promote the expression of interleukin-6 (IL-6) in tumor cells, thereby inhibiting the T-cell immune response (63).

## Modulation of TAMs to Elevate Anti-PD-1/PD-L1 Immunotherapy

**Figure 2:**
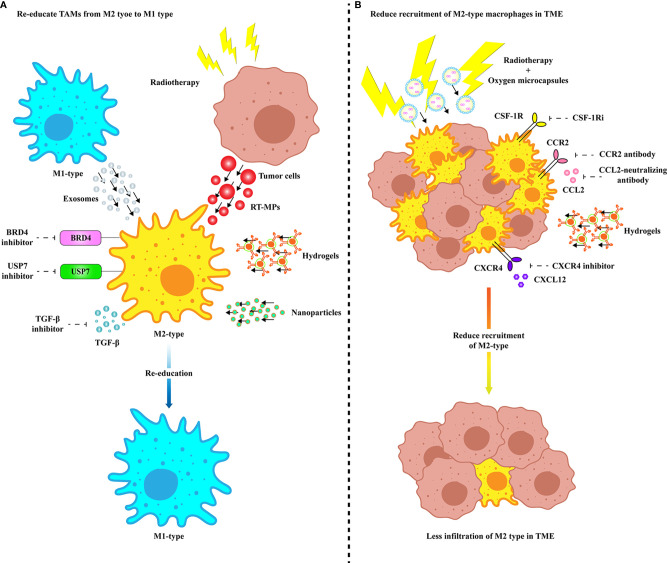
Treatments targeting TAMs to improve anti-PD-1/PD-L1 efficacy. **(A)** Multiple modes of re-education from M2 TAMs to M1 phenotype. Specific inhibition of membrane proteins of M2 TAMs and TGF-β secretion can enable TAMs to obtain tumoricidal phenotype. M2 TAMs are repolarized to M1 type by internalizing vesicles from M1-type macrophages and radiated tumor cells. Precise targeting of TAMs in TME improves the efficacy of anti-PD-1/PD-L1 therapy through drug delivery platforms such as nanovesicles and hydrogels. **(B)** Various ways to inhibit TAM recruitment and infiltration in TME. Infiltration of TAMs in TME can be inhibited by specific inhibition of receptors expressed on the surface of TAM cell membrane or by delivery of drugs *via* hydrogel. Radiotherapy combined with ameliorating the hypoxia microenvironment can effectively eliminate the infiltration of TAMs in TME. BRD4, Bromodomain-containing protein 4; CCL, C-C motif chemokine ligand; CCR, C-C motif chemokine receptor; COX-2, Cyclooxygenase 2; CSF-1R, Colony-stimulating factor 1 receptor; CSF-1Ri, CSF-1R inhibitor; CXCL, CXC-motif chemokine ligand; CXCR, C-X-C chemokine receptor; RT-MP, Microparticles released by radiated tumor cell; TGF-β, Transforming growth factor-β; USP7, Ubiquitin-specific protease 7.

As mentioned above, TAMs have a multichannel inhibitory effect on anti-PD-1/PD-L1 immunotherapy. Therefore, targeting TAMs is of great significance to improve the efficacy of anti-PD-1/PD-L1 immunotherapy. Currently, strategies are designed to deplete TAMs, re-educate TAMs, and intervene with chemokines secreted by TAMs. The combination of the exosomes secreted by M1 macrophages and other nanoimmunotherapy strategies provides more potential options to reduce the occurrence of PD-1/PD-L1 immunosuppression (**Figure 2**). In addition, we summarize the current clinical trials on different targets of TAMs in combination with anti-PD-1/PD-L1 mAbs (**Table 1**).

**Table 1:** Characteristics of clinical trials and drugs on TAM-targeted therapy stratified by targeting mechanisms.

Targeting pathways and mechanisms	Active drugs	Combination therapy	Cancer type	Phase	Clinical Trial ID
CSF-1/CSF-1R	ARRY-382	Pembrolizumab	Advanced Solid Tumors	II	NCT02880371
	Pexidartinib	Durvalumab	Colorectal Cancer, Pancreatic Cancer	I	NCT02777710
CCL2/CCR2	BMS-813160	Nivolumab	Colorectal Cancer, Pancreatic Cancer	Ib/II	NCT03184870
			Non-small Cell Lung Cancer Hepatocellular Carcinoma	II	NCT04123379
			Advanced Cancer	II	NCT02996110
			Pancreatic Ductal Adenocarcinoma	I/II	NCT03767582 NCT03496662
CCL5/CCR5	Maraviroc	Pembrolizumab	Metastatic Colorectal Cancer	I	NCT03274804
	Vicriviroc	Pembrolizumab	Colorectal Neoplasms	II	NCT03631407
CXCL12/CXCR4	Motixafortide	Cemiplimab	Pancreatic Cancer	II	NCT04543071
CD40/CD40L	ABBV-927	Budigalimab	Pancreatic Cancer	II	NCT04807972
	Selicrelumab	Atezolizumab	Solid Tumors	I	NCT02304393
	YH-003	Toripalimab	Advanced Solid Tumors	I/II	NCT04481009 NCT05031494
		Pembrolizumab	Solid Tumor	I	NCT05176509
TLR7	BDB-001	Atezolizumab	Solid Tumor	I/II	NCT03915678 NCT04196530
		Pembrolizumab	Solid Tumor	I	NCT03486301 NCT04840394
	BDC-1001	Nivolumab	HER2 Positive Solid Tumors	I/II	NCT04278144
	DSP-0509	Pembrolizumab	Neoplasms	I/II	NCT03416335
	Resiquimod	Pembrolizumab	Advanced Solid Tumor Locally Advanced Solid Tumor Metastatic Solid Tumor	I/II	NCT04799054
TLR8	SBT6050	Pembrolizumab	HER2 Positive Solid Tumors	I/Ib	NCT04460456
	Motolimod	Durvalumab	Ovarian Cancer	I/II	NCT02431559
TLR9	CMP-001	Atezolizumab	Non-Small Cell Lung Cancer	Ib	NCT03438318
		Nivolumab	Melanoma, Lymph Node Cancer	II/III	NCT04695977 NCT04698187 NCT04401995 NCT03618641
		Pembrolizumab	Carcinoma, Squamous Cell of Head and Neck	II	NCT04633278
			Lymphoma	I/II	NCT03983668
			Melanoma	Ib/II	NCT03084640 NCT02680184 NCT04708418
	Tilsotolimod	Nivolumab	Advanced Cancer	I	NCT04270864
			Solid Tumor	II	NCT03865082
	SD-101	Nivolumab	Pancreatic Adenocarcinoma	I	NCT04050085
			Metastatic Uveal Melanoma in the Liver	I/Ib	NCT04935229
		Pembrolizumab	Prostatic Neoplasms	II	NCT03007732
		Pembrolizumab Nivolumab	Hepatocellular Carcinoma Intrahepatic Cholangiocarcinoma	Ib/II	NCT05220722
	Cavrotolimod	Pembrolizumab Cemiplimab	Advanced or Metastatic Solid Tumors	Ib/II	NCT03684785
TLR3	Rintatolimod	Pembrolizumab	Ovarian Cancer Recurrent	I/II	NCT03734692
PI3Kγ signal pathway	Copanlisib	Durvalumab	Non-Small Cell Lung Cancer	I	NCT04895579
		Nivolumab	Colon Cancer	I/II	NCT03711058
			Malignant Solid Neoplasm	I/II	NCT04317105
			Non-Small Cell Lung Cancer Head and Neck Squamous Cell Carcinoma Hepatocellular Carcinoma	Ib/II	NCT03735628
			Ann Arbor Stage III Lymphoma Ann Arbor Stage IV Lymphoma Solid Neoplasm	Ib	NCT03502733
			Indolent Lymphoma	Ib	NCT04431635
			Lymphoma	I	NCT03884998
			Recurrent Diffuse Large B-Cell Lymphoma	II	NCT03484819
	Duvelisib	Nivolumab	Unresectable Melanoma	I/II	NCT04688658
			Chronic Lymphocytic Leukemia Diffuse Large B-Cell Lymphoma	I	NCT03892044
CD47/SIRPα pathway	ALX-148	Pembrolizumab	Microsatellite Stable Metastatic Colorectal Cancer	II	NCT05167409
			Solid Tumor, Non-Hodgkin Lymphoma	I	NCT03013218
			Head and Neck Cancer Head and Neck Squamous Cell Carcinoma	II	NCT04675294 NCT04675333
	AO-176	Pembrolizumab	Solid Tumor	I/II	NCT03834948
	Magrolimab	Pembrolizumab	Head and Neck Squamous Cell Carcinoma	II	NCT04854499
			Hodgkin Lymphoma	II	NCT04788043
STING pathway	SNX-281	Pembrolizumab	Advanced Solid Tumor Advanced Lymphoma	I	NCT04609579
	BMS-986301	Nivolumab	Advanced Solid Cancers	I	NCT03956680
	SYNB-1891	Atezolizumab	Metastatic Solid Neoplasm, Lymphoma	I	NCT04167137
	TAK-676	Pembrolizumab	Solid Neoplasms	I	NCT04420884
	MK-2118	Pembrolizumab	Solid Tumor, Lymphoma	I	NCT03249792
	MK-1454	Pembrolizumab	Solid Tumors, Lymphoma	I	NCT03010176
			Head and Neck Squamous Cell Carcinoma	II	NCT04220866
	SB-11285	Atezolizumab	Solid Tumor, Melanoma Head and Neck Squamous Cell Carcinoma	Ia/Ib	NCT04096638

### Depletion of M2 TAMs

As the infiltration of TAMs limits clinically relevant immune responses (64–66), depleting TAMs seems to be an attractive strategy. Colony-stimulating factor 1 receptor (CSF-1R) blockers are the main method by which to deplete M2 TAMs (67, 68). CSF-1 binds to its receptor, and the latter then undergoes autophosphorylation, which plays an important role in the proliferation, differentiation, and maintenance of macrophages (69). Therefore, a CSF-1R inhibitor (CSF-1Ri) may improve the efficacy of anti-PD-1/PD-L1 immunity. PLX3397 (pexidartinib), a CSF-1R kinase inhibitor, can increase the infiltration and antitumor function of CD8^+^ T cells in tumors when combined with anti-PD-1 therapy (70, 71). At the same time, it can also effectively reduce the appearance of tumor neovascularization and ascites (72). BLZ945, another CSF-1Ri, combined with a PD-1/PD-L1 blocking antibody is also effective in controlling tumor growth (13, 73). Inherent antitumor drugs can also target CSF-1R to provide a new method for combined immunotherapy. Erlotinib, a first-generation small-molecule inhibitor targeting the epidermal growth factor receptor (EGFR) tyrosine kinase, is suitable for the first-line treatment of EGFR mutation-positive non-small cell lung cancer (NSCLC). Combination therapy consisting of its derivative TD-92 and anti-PD-1 contributes to reduced tumor growth and increased survival *in vivo* (74).

### Re-Education of M2 TAMs

Recently, several findings have suggested that re-education of M2 TAMs rather than depletion may represent a more effective strategy. Previous studies have reported that the protumor M2 phenotype can be re-educated to the tumoricidal M1 phenotype, thereby inhibiting the supporting role of TAMs in tumors (75). BRD4, a bromodomain and extraterminal (BET) family protein, can enhance the expression of CCL2 by activating the NF-κB signaling pathway, which, in turn, causes the recruitment of macrophages in tumors (76). The BRD4 inhibitor AZD5153 can re-educate TAMs from M2 to M1 and promote the secretion of proinflammatory cytokines, thereby activating cytotoxic T lymphocytes (CTLs) *in vitro* (77). More importantly, AZD5153 was proven to render ovarian cancer sensitive to anti-PD-L1 treatment through a 3-D microfluidic model (77). SF2523, another BRD4 inhibitor that can block the polarization of TAMs, restores the activity of CD8^+^ T cells and then stimulates the antitumor immune response (78). Furthermore, the BET inhibitor JQ1 can significantly reduce PD-L1 expression on tumor cells and TAMs and limit tumor progression in a cytotoxic T-cell-dependent manner (79).

Ubiquitin-specific protease 7 (USP7), a deubiquitinating enzyme, is considered a promising therapeutic target because of its regulatory role in DNA damage and epigenetic inheritance (80). USP7 has been identified as a highly expressed M2 TAM gene, and specific inhibition of USP7 can reprogram M2 TAMs into M1 through the P38 MAPK pathway (81). In addition, targeting USP7 promoted the infiltration and cytotoxicity of CD8^+^ T cells in the TME and decreased PD-L1 expression in tumor cells (81, 82).

### Inhibition Chemokines Secreted by M2 TAMs

In addition to targeting M2 TAMs themselves, the immunosuppressive effects of M2 TAMs can also be reversed by inhibiting cytokines secreted by M2 TAMs.

As one of the most important immunosuppressive cytokines secreted by M2 TAMs, TGF-β changes the TME by limiting the infiltration of T cells to inhibit antitumor immunity (8). Combination therapy with a TGF-β inhibitor (1D11 and galunisertib) and anti-PD-1/PD-L1 resulted in the upregulation of immune response genes, restoring the cytotoxic activity of T cells and the antitumor activity of anti-PD-L1 (32, 83). In addition to the above effects, combinatorial treatment consisting of tranilast, a TGF-β inhibitor, with Doxil nanomedicine has been shown to improve M1 macrophage content in the tumor tissue, which results in the increased efficacy of anti-PD-1 (84). Phase II research is ongoing to investigate the combined effect of TGF-β inhibitor (vactosertib) with anti-PD-L1 (durvalumab) (NCT04064190), while another phase II study is assessing the efficacy and safety of NIS793 with and without spartalizumab (NCT04390763) in untreated metastatic pancreatic ductal adenocarcinoma (mPDAC). In addition, M7824 is a bifunctional fusion protein comprising a mAb against PD-L1 fused to the extracellular domain of TGF-β receptor 2 (85). Compared with targeting TGF-β alone, M7824 has been proven to reduce tumor burden and improve overall survival (OS) (85). A phase II trial was performed to determine the efficacy of M7824 plus topotecan or temozolomide in recurrent SCLC (NCT03554473).

CCL2 is a chemokine that attracts a number of CCR2-high-expressing monocytes to the tumor site. The role of C-C motif chemokine receptor 2 (CCR2) seems to influence TAM recruitment at the tumor site. In a recent study, it was proven that CCR2 is involved in the recruitment and initiation of tumor-promoting inflammation (86). Many preclinical studies showed high efficacy of CCL2/CCR2 antagonists; for instance, targeting CCR2 with a small-molecule inhibitor not only reduced recruitment of M2-type macrophages but also induced tumor infiltration of activated CD8^+^ T cells (87). Many other preclinical studies on different tumor types showed that either depletion of CCR2 or disruption of the CCL2-CCR2 interaction has an impact on the inhibition of TAM recruitment and tumor regression or inhibition of metastasis (88–91). A phase I/II trial of combination immunotherapy with nivolumab and a CCR2/CCR5 dual antagonist (BMS-813160) is in progress to evaluate whether this therapy is safe in patients with locally advanced pancreatic cancer (LAPC) (NCT03767582).

CXC-motif chemokine ligand 12 (CXCL12) is another chemokine that regulates the migration of monocytes (92). Elevated C-X-C chemokine receptor 4 (CXCR4) is correlated with the tumorigenesis of NSCLC (93). CXCL12 secretion could be induced in response to radiation therapy and cause the accumulation of TAMs in the tumor (94). BL-8040 (motixafortide), one of the CXCR4 antagonists, plus the anti-PD-1 pembrolizumab in the COMBAT trial contributes to the improvement found in pancreatic ductal adenocarcinoma (PDAC) patients (95). After using a new CXCR4 inhibitor peptide R, the expression of CD73, CD38, and IL-10 in non-small cell lung cancer is reduced, which can rescue the cytotoxic activity of T cells and prevent TAM polarization (96). Another CXCR4 antagonist, Pep R, demonstrated efficacy in combination with nivolumab in melanoma. In addition, there are already 2 observational studies in progress to study whether Pep R can reverse anti-PD1 resistance (NCT03891485 and NCT03628859).

Many people have recognized the potential of TGF-β, and there are currently many clinical trials combining TGF-β targeting with PD-1/PD-L1 treatment (97). However, most studies have stalled due to serious adverse events or the observation of minimal clinical benefit (98), which may be associated with higher global drug levels. As CXCR4 is widely expressed in hematopoietic cells and a variety of stem cells (99), therapies targeting CXCR4 face a similar dilemma. In contrast, therapies that block the CCL2–CCr2 axis appear to be safer and are expected to elucidate the mechanism of action in different cancer species. In addition, for the current targeted drugs yielding a poor systemic response, the combination of cell-targeted drug delivery systems is an ideal choice to counteract their limitations.

### Nanoimmunotherapy Strategies

Compared with traditional delivery systems, nanoparticles that can specifically deliver drugs to TAMs and modulate their polarized states may be an effective method in cancer immunotherapy. For example, tumor cell-derived microparticles containing the chemotherapeutic drug methotrexate (TMP-MTX), nanoparticles delivering shikonin and PD-L1 knockdown siRNA (SK/siR-NPs), and Gadofullerene (GF-Ala) nanoparticles can all reprogram M2 TAMs to an M1-like phenotype and increase the infiltration of CTLs, thereby effectively inhibiting tumor growth (100–102). CMPB90-1, a new natural polysaccharide from *Cordyceps*, converts immunosuppressive TAMs by binding to toll-like receptor 2 (TLR2), polarizes TAMs to the M1 phenotype, and has antitumor effects and a better safety profile (103). These studies may provide a promising strategy for the development of high-efficiency, low-toxicity immunotherapy based on nanotechnology.

Due to the unpredictable toxicity and poor scalability of nanocarriers in the human body (104), other advanced drug carriers have gradually attracted attention, such as hydrogels (105), exosomes (106), and enucleated cells (107). A Melittin-Rada32 hybrid peptide hydrogel loaded with doxorubicin (DOX) was designed to reshape the tumor immune microenvironment in the treatment of melanoma, which specifically consumes M2 TAMs and increases activated CTL infiltration (108). Dai et al. designed a hydrogel scaffold loaded with KN93, a Ca^2+^/calmodulin-dependent protein kinase II (CAMKII) inhibitor, which can reprogram TAMs into the M1 phenotype (109). After this hydrogel treatment, CTL infiltration in the TME increased, and the expression of macrophage PD-L1 increased, suggesting that it has good prospects for anti-PD-1 treatment (109).

### Modification of Exosomes Derived From M1 Macrophages

Studies have reported that exosomes derived from macrophages have immunomodulatory effects (110). Exosomes secreted by M1 macrophages are reported to inhibit the development of gastric cancer and activate T-cell-dependent immune responses (111). Endogenous macrophage exosomes have been shown to have absolute advantages over their safety (112). Therefore, M1 macrophage-derived exosomes (M1-exos) can be used to deliver various anticancer drugs for tumor therapy. Mannose-modified macrophage-derived microparticles (Man-MPs) loaded with metformin have been developed to efficiently target M2-like TAMs to repolarize them into the M1-like phenotype (113). More importantly, the collagen-degrading capacity of Man-MPs contributes to the infiltration of CD8^+^ T cells into tumor interiors and enhances tumor accumulation and penetration of anti-PD-1 (113). Macrophage-derived exosomes loaded with PTX and Dox were developed to treat triple-negative breast cancer (TNBC) *in vivo* (114). With the development of nanotechnology, exosome-mimetic nanovesicles derived from M1 macrophages (M1NVs) were designed to repolarize M2 TAMs to M1 macrophages (115). Moreover, injection of a combination of M1NVs and anti-PD-L1 further reduced the tumor size compared with the injection of either M1NVs or aPD-L1 alone (115).

### Radiotherapy

As a conventional means of tumor treatment, radiotherapy leads to increased expression of PD-L1 in tumor cells (116), which is one of the markers of anti-PD-1/PD-L1 mAb therapy (117). Interestingly, in a model of malignant pleural effusions, microparticles released by radiated tumor cells (RT-MPs) can precisely locate M2-TAMs in the TME and convert the latter into M1-TAMs by activating the JAK-STAT and MAPK pathways (118). It is noteworthy that the combination of RT-MPs and anti-PD-1 exhibits good biocompatibility and memory immune response, which TMP-MTX cannot match (118). Since hypoxia has been proven to be an important factor affecting the clinical outcome after radiotherapy (119), strategies to interfere with hypoxia have been developed to optimize radiotherapy (120). Oxygen microcapsules are designed to rapidly increase the oxygen concentration in the TME, resulting in reduced infiltration of TAMs while enhancing the efficacy of radiotherapy (121). Of particular note is the microcapsules’ ability to repolarize TAMs into the M1 phenotype, which, in turn, activates the T-cell-mediated antitumor immune response (121). Obviously, this nanotherapy can further enhance the efficacy of radiotherapy combined with anti-PD-1/PD-L1 mAbs.

## Future Perspectives

The application of ICIs in clinical practice has completely changed the therapeutic strategies for cancer patients by improving the prognosis and reducing the impact on their quality of life (QoL) compared with standard approaches (117, 122–129). However, the high cost of anti-PD-1 and PD-L1 agents (130, 131) highlights the need to select patients who will benefit most from the treatment early, supporting the research on predictive biomarkers of response and strategies to overcome resistance and optimize the efficacy of these drugs.

Targeting macrophages to treat cancer is a young but rapidly developing area of research and therapy. Despite great interest, the optimum therapeutic approach has not yet been identified because TAMs represent a heterogeneous population, and their role in tumors varies depending on many environmental conditions. The other difficulty arises from the TME, which is a very dynamic tissue and contains various infiltrating immune cells and external factors that influence tumor progression, macrophage polarization, and therapeutic response. Some macrophage-targeting therapeutics are effective as monotherapies. However, more evidence exists that targeting TAMs could improve the efficacy of conventional therapies and immune therapeutics. Currently, two main approaches that target TAMs with apparent opposite effects have been developed. One approach is to deplete macrophages, and the other is to re-educate them to kill cancer cells. Depending on the macrophage infiltration status and the chosen therapy as a combination treatment, various approaches will be chosen. For example, through their Fcγ receptors, macrophages were shown to take up therapeutic antibodies such as anti-PD-L1, limiting the efficacy of such therapeutic modalities in animal models. In fact, in several recent studies, it was shown that depleting macrophages with the use of CCL2/CCR2 antagonists improves the efficacy of PD-L1-targeting antibodies and possibly other ICIs (132, 133).

In addition, some important issues should be resolved before TAM antagonists are used to overcome resistance to immunotherapy. More convincing clinical studies are needed to confirm the correlation of macrophage infiltration or phenotype with the outcomes of patients under anti-PD-1/PD-L1 therapy. It is essential to identify subpopulations that have the potential to benefit from different therapies targeting macrophages. Despite these difficulties, there is still great potential to harness macrophage biology to improve the efficiency of anti-PD-1/PD-L1 ICIs in oncology.

## Author Contributions

YP and QJ conceived the structure of manuscript and revised the manuscript. YP created the figures. QJ reviewed the manuscript. All authors contributed to the article and approved the submitted version.

## Funding

This work was supported by the National Science Foundation of China (82074225 to QJ).

## Conflict of Interest

The authors declare that the research was conducted in the absence of any commercial or financial relationships that could be construed as a potential conflict of interest.

## Publisher’s Note

All claims expressed in this article are solely those of the authors and do not necessarily represent those of their affiliated organizations, or those of the publisher, the editors and the reviewers. Any product that may be evaluated in this article, or claim that may be made by its manufacturer, is not guaranteed or endorsed by the publisher.

## Abbreviations

AKT: Protein kinase B; BET: Bromodomain and extraterminal; BRD4: Bromodomain-containing protein 4; CAMKII: Ca^2+^/calmodulin-dependent protein kinase II; CCL: C-C motif chemokine ligand; CCR: C-C motif chemokine receptor; COX-2: Cyclooxygenase 2; CSF-1R: Colony-stimulating factor 1 receptor; CSF-1Ri: CSF-1R inhibitor; CTL: Cytotoxic T lymphocyte; CTLA-4: Cytotoxic T lymphocyte antigen 4; CXCL: CXC-motif chemokine ligand; CXCR: C-X-C chemokine receptor; DC: Dendritic cell; EGFR: Epidermal growth factor receptor; Foxp3: Forkhead Box P3; GF-Ala: Gadofullerene; ICI: Immune checkpoint inhibitor; IL: Interleukin; LAPC: Locally advanced pancreatic cancer; M1-exo: M1 macrophage-derived exosome; M1NV: Nanovesicle derived from M1 macrophages; mAb: Monoclonal antibody; Man-MP: Mannose-modified macrophage-derived microparticle; miR: MicroRNA; mPDAC: Metastatic pancreatic ductal adenocarcinoma; mPGES1: Microsomal PGE2 synthase1; NF-κB: Nuclear factor kappa-B; NK cell: Natural killer cell; NSCLC: Non-small cell lung cancer; OS: Overall survival; PDAC: Pancreatic ductal adenocarcinoma; PD-1: Programmed cell death 1; PD-L1: Programmed cell death ligand-1; PGE2: Prostaglandin E2; PI3K: Phosphatidylinositol 3-kinase; QoL: Quality of life; RT-MP: Microparticles released by radiated tumor cell; SK: Shikonin; SK/siR-NP: Versatile nanoparticle codelivering SK and PD-L1 knockdown siRNA; TAM: Tumor-associated macrophage; TGF-β: Transforming growth factor-β; TLR: Toll-like receptor; TME: Tumor microenvironment; TMP-MTX: Tumor cell-derived microparticles containing the chemotherapeutic drug methotrexate; TNBC: Triple-negative breast cancer; Treg: Regulatory T cell; USP7: Ubiquitin-specific protease 7; VISTA: V-domain Ig-containing suppressor of T-cell activation.

## Glossary

AKT: Protein kinase B
BET: Bromodomain and extraterminal
BRD4: Bromodomain-containing protein 4
CAMKII: Ca^2+^/calmodulin-dependent protein kinase II
CCL: C-C motif chemokine ligand
CCR: C-C motif chemokine receptor
COX-2: Cyclooxygenase 2
CSF-1R: Colony-stimulating factor 1 receptor
CSF-1Ri: CSF-1R inhibitor
CTL: Cytotoxic T lymphocyte
CTLA-4: Cytotoxic T lymphocyte antigen 4
CXCL: CXC-motif chemokine ligand
CXCR: C-X-C chemokine receptor
DC: Dendritic cell
EGFR: Epidermal growth factor receptor
Foxp3: Forkhead Box P3
GF-Ala: Gadofullerene
ICI: Immune checkpoint inhibitor
IL: Interleukin
LAPC: Locally advanced pancreatic cancer
M1-exo: M1 macrophage-derived exosome
M1NV: Nanovesicle derived from M1 macrophages
mAb: Monoclonal antibody
Man-MP: Mannose-modified macrophage-derived microparticle
miR: MicroRNA
mPDAC: Metastatic pancreatic ductal adenocarcinoma
mPGES1: Microsomal PGE2 synthase1
NF-κB: Nuclear factor kappa-B
NK cell: Natural killer cell
NSCLC: Non-small cell lung cancer
OS: Overall survival
PDAC: Pancreatic ductal adenocarcinoma
PD-1: Programmed cell death 1
PD-L1: Programmed cell death ligand-1
PGE2: Prostaglandin E2
PI3K: Phosphatidylinositol 3-kinase
QoL: Quality of life
RT-MP: Microparticles released by radiated tumor cell
SK: Shikonin
SK/siR-NP: Versatile nanoparticle codelivering SK and PD-L1 knockdown siRNA
TAM: Tumor-associated macrophage
TGF-β: Transforming growth factor-β
TLR: Toll-like receptor
TME: Tumor microenvironment
TMP-MTX: Tumor cell-derived microparticles containing the chemotherapeutic drug methotrexate
TNBC: Triple-negative breast cancer
Treg: Regulatory T cell
USP7: Ubiquitin-specific protease 7
VISTA: V-domain Ig-containing suppressor of T-cell activation
